# Recent Advances in the Diagnosis and Management of Congenital Heart Defects

**DOI:** 10.3390/jcm11195534

**Published:** 2022-09-21

**Authors:** Vlasta Fesslova, Paolo Ivo Cavoretto

**Affiliations:** 1 Center of Fetal Cardiology, IRCCS Policlinico San Donato, Via Morandi 30, San Donato Milanese, 20097 Milan, Italy; 2 Gynecology and Obstetrics Department, IRCCS San Raffaele Hospital, University Vita-Salute, Via Olgettina 60, 20132 Milan, Italy

The prenatal assessment of congenital heart defects (CHD) and related fetal and maternal management is very challenging and delicate. Therefore, all operators/specialists involved in fetal cardiology will benefit from the more recent updates in different fetal situations. In this Special Issue of the Journal of Clinical Medicine titled “Prenatal diagnosis and management of CHD”, different novel aspects of prenatal diagnostics were reported.

The first topic of interest that we would like to highlight is related to fetal arrhythmias. Veduta et al. [1] presented an original review on the treatment of this condition, wherein they distinguished the benign forms—in which fetal surveillance and monitoring are recommended with no specific treatment required—from those more serious, in which the physicians should undertake specific treatments while the fetus is still in utero. The authors concluded that a majority of fetal tachycardias can be resolved or controlled by transplacental or direct administration of anti-arrhythmic drugs. In contrast, the persistent fetal bradycardia found in structurally normal hearts is mostly due to an atrioventricular block, often caused by anti Ro/SSA and anti La/SSB antibodies, and in this condition the efficacy of prenatal treatment by steroids or eventually sympaticomimetics remains controversial. This paper will be useful for all clinicians interested in the current standard of management of specific types of arrhythmias.

The first trimester diagnosis of CHD is reported in another paper of this Special Issue, in which Herghelegiu and coworkers showed the usefulness of a few ultrasound patterns showing simple abnormalities in the four-chamber, three-vessel, and tracheal views of the fetal heart and described their association with specific CHD types [2]. The authors described five patterns for the four-chamber view and five patterns for the three-vessel and tracheal views, leading to several different categories and associations with a normal heart or with specific cardiac abnormalities. This is an original and clinically useful approach that summarizes different features obtained in two simple standard views and it will certainly be helpful for the examination of small fetuses, which is currently increasingly common in many centers.

The risk of CHD and other anomalies in Assisted Reproduction Technology (ART) pregnancies, compared with spontaneous conceptions, was widely debated and produced controversial results. Recently, a major work from our group showed that the prevalence of CHD after conception with (ART) vs. spontaneous conception is increased by about 50% [3]. Herein, we present new data from a novel cohort from Italy, confirming this experience and showing that, unfortunately, these fetuses present a preponderance of major vs. minor CHD [4]. This knowledge should be taken into account when managing ART pregnancies. Given the increasing use of ART conceptions in different countries, every study concerning possible related problems will be very important for clinical use and a call for future research on this topic should be raised.

A very specific new diagnostic approach was presented in another paper by Sandrini et al. The study assesses the importance of a virtual cardiac autopsy by a MicroCT scan, which can provide a confirmation of the cardiac anatomy of small fetuses, which is otherwise not obtainable by the routine pathological examination due to the small size of these fetal hearts. This paper documents the relative features of certain specific cardiac defects and should be carefully considered as it presents great potential towards wide clinical implementation [5].

The ultrasound investigation of the fetal heart and major vessels in the mediastinum generally does not involve an extensive assessment of other central veins, besides the inferior and superior vena cava. Abnormalities of the left brachiocephalic vein were shown in a few recent papers and inconsistent conclusions were reported with respect to the possible association with other cardiac or extracardiac anomalies. Our group carried out a systematic review of the literature, including some cases from our experience, achieving a remarkable group of more than 300 abnormalities of this vessel from 16 studies that distinguish the types of the left brachiocephalic vein according to its course [6]. The incidence of abnormalities of the left brachiocephalic veins was estimated to be about 0.4% from six cohort studies and neonatal outcomes appeared favorable in most cases, particularly in intrathymic forms. In this review, extracardiac anomalies were found in in 3.5% of cases whereas CHD were associated with 75% of cases with such an anomaly; however, the vast majority of CHD were only minor defects (a persistent left superior vena cava in the case of absent left brachiocephalic veins), while major CHD were always associated with double, retroesophageal, and subaortic forms. This paper proposes introducing the evaluation of this aspect into the routine obstetrical/echocardiographic investigation in order to detect or exclude potential associated cardiac or extracardiac anomalies.

The features of the fetal heart in the rare condition of lymphangioma were described in an interesting paper from Kordjalik et al. [7]. The lymphangiomas in the analyzed series coexisted with CHD in 78% of cases of which two-thirds included structural and one-third functional abnormalities. Almost 40% of the fetuses were genetically abnormal, leading to a very low rate of survival for these fetuses (in 15% of 35% of the remaining cases). This paper emphasizes the importance of genetic studies and the prompt referral to tertiary centers for cases affected by this rare condition, stressing further investigation and management aimed at maximizing survival.

The study from Strzelecka et al. reports a particular epidemiological aspect related to a very serious cardiac anomaly, namely, a hypoplastic left heart, for which the authors found an interesting relationship with air pollution in certain regions of Poland involving the impact of benzopyrene and/or PM10 (Particulate matter of 10 microns or less). The authors also comment on the variable frequency of this cardiac anomaly in different seasons in Poland. This report on possible novel teratogenic mechanisms calls for future studies to further explore this topic [8].

Finally, Kovacevic et al. investigated the impact of the COVID-19 pandemic on parental counselling in fetal cardiac services in Germany. The authors demonstrated that the COVID-19 breakout altered parental perceptions and influenced the effectiveness of counseling for fetal CHD. The authors suggest implementing alternative and innovative approaches such as online conferences or virtual reality tools in order to facilitate high-quality services in critical times such as the present pandemic [9].

Overall, the articles of this Special Issue contribute to a valuable, up-to-date summary of the emerging novel aspects related to cardiac problems in fetal medicine and will likely stimulate other similar studies and research on this topic.
